# Mid-upper arm circumference as a substitute of the body mass index for assessment of nutritional status among adult and adolescent females: learning from an impoverished Indian state

**DOI:** 10.1016/j.puhe.2019.09.010

**Published:** 2020-02

**Authors:** A. Das, G. Saimala, N. Reddy, P. Mishra, R. Giri, A. Kumar, A. Raj, G. Kumar, S. Chaturvedi, S. Babu, S. Srikantiah, T. Mahapatra

**Affiliations:** CARE India Solutions for Sustainable Development, H No. 14, Patliputra Colony, Patna, Bihar, 800013, India

**Keywords:** Anthropometry, Body mass index, Mid-upper arm circumference, Malnutrition, Women's health

## Abstract

**Objectives:**

For population-level screening of malnutrition among adults—especially in developing-country settings—the body mass index (BMI) can be impractical because of logistical requirements for weight and height measurement. We analyzed anthropometric data collected from a large-scale nutritional survey on women of rural Bihar to determine the mid-upper arm circumference (MUAC) cutoffs corresponding to standard BMI cutoffs and the predictive accuracies of the determined cutoffs.

**Study design:**

It was a cross-sectional study using multistage cluster sampling.

**Methods:**

The current analysis used anthropometric data from a study on dietary practices of rural women (adolescents, lactating mothers, and women in the interpregnancy period). The MUAC (cm) cutoffs corresponding to four standard BMI (kg/m^2^) values were determined using receiver operating characteristic (ROC) curve analysis.

**Result:**

We detected a significant positive correlation between BMI and MUAC (r = 0.81, *P* < 0.0001). In ROC curve analysis, the MUAC cutoffs corresponding to BMI cutoffs of 18.5, 23, 25, and 30 kg/m^2^ were estimated to be 23.2, 26.0, 27.3, and 30.5 kg/m^2^, respectively. The predictive accuracy of the determined cutoffs was good, as indicated by the area under the ROC curve for the four different cutoffs—which ranged between 88% and 97%. Other than the cutoff for ‘obese’ (BMI, 30 kg/m^2^), the *Kappa* coefficients for the rest of the MUAC cutoffs showed ‘substantial’ agreement (>0.6) with their BMI counterparts.

**Conclusion:**

The results suggest that the cutoffs based on MUAC—a less resource-intensive measure than BMI—can be used for community-based screening of malnutrition among women of Bihar.

## Highlights

• Body mass index (BMI) is a widely used metric for assessing nutritional status among adults.
• However, it is difficult to assess in community settings because of logistical and technical challenges.
• Mid-upper arm circumference (MUAC) can be used in lieu of BMI.
• The present study detected the MUAC values corresponding to the standard BMI cutoff among rural Indian women.

## Introduction

Nutrition is one of the key determinants of the quality of life both among children and adults.^1^ Malnourishment among adult women is associated with a variety of subsequent illnesses that lead to increased risk of morbidity and mortality and affect the country's economy by increasing the burden on state-funded and out-of-pocket expenditure and also by affecting productive life years.^2^, ^3^, ^4^ From public health perspective, nutritional status of adolescent and adult women, especially those of reproductive age, is an important parameter to assess the overall health status of a nation.^5^ However, only a few community-based studies in India have assessed the nutritional status of adult women as most of such studies have focused on ‘infants and young children’.^6^

Various anthropometric measurement techniques are used to assess nutritional status such as body mass index (BMI), mid-upper arm circumference (MUAC), measurement of the thickness of triceps or subscapular skinfolds, and calf circumference.^7^ Although these anthropometric assessments are considered to be less reliable for assessing malnutrition than sophisticated but expensive body composition assessment techniques such as hydrodensitometry, electronic bioimpedance, dual-energy X-ray absorptiometry, and so on,^8^, ^9^, ^10^ simplicity of usage and low cost of implementation make these assessments the ideal choice for population-based evaluations.^10^^,^^11^ BMI, a marker for generalized adiposity and measured as body weight (in kg) divided by height (in meter^2^) squared, is the most widely used anthropometric measure as it is inexpensive and non-invasive and can be collected by evaluators with minimal training.^12^^,^^13^ Therefore, assessment of BMI became popular not only as individual-level clinical and nutritional assessments but also as a survey tool, especially for assessment of undernutrition in developing nations.^13^^,^^14^ A BMI of <18.5 kg/m^2^ is widely accepted as a sign of chronic energy deficiency, where the energy intake equals the energy expenditure, regardless of body weight and body energy stores.^15^ Moreover, prior research studies suggest that BMI, besides being a sensitive marker for nutritional deficiencies/surplus and a crucial determinant of morbidities associated with malnutrition,^16^ can also serve as a surrogate for the socio-economic status of a community.^13^^,^^14^^,^^17^ Nevertheless, in large-scale population-based surveys and for regular monitoring, assessment of BMI is often impractical because of logistical reasons as the equipment for assessment of weight and height often proves unwieldy in the field. Moreover, it is difficult to measure weight and height for non-ambulatory participants/patients.

Under these circumstances, MUAC, a popular anthropometric measure for assessing the nutritional status of children younger than 5 years, has been suggested as an alternative for nutritional status evaluation of adults as well—especially in resource-limited settings.^18^^,^^19^ MUAC has long been used as a tool for anthropometric measurement as it is easier to implement than BMI, with minimum requirement of equipment and acceptable sensitivity and specificity for detecting underweight.^19^^,^^20^ During recent times, MUAC has been used for evaluation of adult nutritional status as well, especially in resource-limited settings, including India.^11^^,^^16^^,^^18^^,^^20^, ^21^, ^22^ Prior studies also suggest that MUAC can be an efficient indicator of adult undernutrition—comparable or even better than BMI.^16^^,^^23^ Thus, given its simplicity and attribute of being less resource intensive, MUAC could be an ideal choice for community-based assessment of undernutrition among rural Indian women. Against this background, the present study sets out to determine the MUAC cutoffs equivalent to BMI cutoffs among adolescent and married women of reproductive age in Bihar.

## Methods

### Study objectives

The Concurrent Measurement and Learning unit of CARE India's Bihar Technical Support Program conducted the ‘Women's Nutrition Study’ in August–September 2016 with the following key objectives:

• To assess the adequacy of food and nutrient (both macronutrients and micronutrients including dietary diversity) intake among women
• To estimate the coverage of iron folic acid supplementation and anthelmintics and determine consumptions of the same
• To evaluate the anthropometric indicators in the study population
• To understand the knowledge level and practices regarding various women's nutritional parameters

### Study design

The present study used a cross-sectional design implemented using multistage cluster sampling.

### Study sample

The study collected data from the following groups of women (representative of women of rural Bihar)—(1) pregnant; (2) lactating; (3) non-pregnant and non-lactating women (women in the interpregnancy period [WIPP]); and (4) adolescent women (married or unmarried) who did not belong to any of the aforementioned categories. The four groups of participants were decided based on the four key beneficiary groups under the current nutrition program: adolescent women, pregnant women, lactating women, and also WIPP (as these women are likely to be pregnant again). Given the low age of marriage and first pregnancy in rural Bihar,^24^ the sampled adolescents were also expected to be representative of the women who have never been pregnant. The selection criteria of different groups of women were as follows:

**Table:** Selection criteria for the different groups of women.

Pregnant women	Lactating women	Non-pregnant, non-lactating women	Adolescent women
• In the 2nd or 3rd trimester of pregnancy • Adult (≥18 years)	• Biological mothers of up to 6-month-old living children • Ever breastfed the last born child • Adult (≥18 years)	• Mothers who have had at least one live birth and intend to have another child • Adult (≥18 years)	• Between 15 and 17 years old (married or unmarried), lowest age encountered in the sample was 15 years • Non-pregnant and non-lactating

To ensure statewide representativeness, respondents were selected from all 534 blocks (subdistricts) in Bihar. For within-block sampling, a list of Anganwadi centers (AWCs)—village-level institutions providing basic healthcare services—was used. From each block, two AWC catchment areas were selected by cluster random sampling. This sampling strategy resulted in selection of 1068 (534*2) AWC catchment areas across Bihar. In the selected AWC catchment areas, one index household was selected using a random number table. Then, following a ‘Right-hand (clockwise)’ rule (and excluding first five households from the index household), the enumerators went around the village until they came across a household containing an eligible, consenting participant from any of the four target groups. Once a successful interview was conducted, the next five households were excluded to lower possible neighborhood effect regarding knowledge/behavior, and search for next eligible participant was continued using the ‘Right-hand’ rule. As many of the nutritional parameters and proximal outcomes such as knowledge may depend largely on the quality of services provided by the designated Anganwadi workers (AWWs), only a single participant from each group was interviewed in each of the selected AWC catchment areas to minimize the effect of intracluster correlation in the overall sample.

### Measurements

From each of the sampled AWC catchment areas, interviews on dietary intake pattern and food availability were conducted with one eligible participant belonging to each of the four categories, resulting in 1068 respondents per category. For selection of eligible women from within the selected AWC catchment areas, a systematic sampling methodology similar to that used in earlier studies^25^^,^^26^ was used.

Standard anthropometric measurements—standing height and weight—were carried out for approximately 25% of the consenting respondents (25% from each target group—the AWC catchment areas for measurement were selected beforehand using simple random sampling). MUAC was measured for all participants. In the subsample selected for anthropometric measurement, if the interview was not completed (or if the participant provided consent for interview only but not for measurement), then a replacement interview was conducted in the same village with a participant belonging to the same target group. Standing height, weight, and MUAC were measured using portable stadiometers (SECA code 213), digital weighing flat scales (SECA code 874), and non-stretchable measuring tapes, respectively. The weighing machine was calibrated to measure differences of up to 10 g, whereas one millimeter was the minimum measurement possible for height and MUAC. For measuring height, it was ensured that the participant neither was wearing any footwear/socks nor had any buns/hair ornaments. The participants were instructed to stand with the back of their head, scapula, buttocks, and back of heels making contact with the back plate of the stadiometer and the toes pointing outward. The head was positioned so that the infraorbital ridge and upper border of the external auditory meatus was in the same horizontal plane (Frankfort's plane). During weight measurement, the participants were requested to remove any heavy removable items/clothing/shoes, as far as culturally appropriate, and stand on the center of the weighing machine with hands at their sides and looking straight ahead. MUAC measurement was performed in the left arm at the midpoint of the acromion process and olecranon process. Each of the anthropometric measurement was conducted twice, and both the values were recorded. At the analysis level, the arithmetic mean of the two measures was calculated. Other than pregnant women, anthropometric data from the rest of the three categories of women were used for the current analysis.

Overall, 310 enumerators, who underwent standardized training in different batches, conducted data collection and MUAC measurements. From these enumerators, 80 were selected for further training on anthropometric assessments, i.e., assessment of height and weight.

### Statistical analysis

Descriptive analyses were carried out to determine the distribution of sociodemographic and anthropometric characteristics of the study population. The nature of relationship (linear/curvilinear) between BMI and MUAC was assessed using Box–Cox transformation and identity function (Transreg procedure in SAS). After establishment of a linear relationship (based on the ‘lambda’ value), we performed Pearson's correlation between BMI and MUAC to assess if a statistically significant positive or negative association existed between these two variables. Simple linear regression analysis was performed to determine the strength of association (and statistical significance) between BMI and MUAC. The MUAC values corresponding to four standard BMI cut points,^27^ namely, 18.5, 23, 25, and 30 kg/m^2^, were determined using receiver operating characteristic (ROC) curve analysis. Three methods were used to estimate the optimal cutoff values from the ROC curves: (1) Youden's *J* statistic; (2) minimized distance to the (0,1) point in the ROC curve; and (3) sensitivity-specificity equality.^28^, ^29^, ^30^, ^31^ In case of discordance between these three methods, the cutoff value determined by Youden's *J* statistic was chosen. Furthermore, the predictive accuracy of each cutoff point for MUAC was assessed by determining the sensitivity, specificity, and total misclassification percentage against the corresponding BMI cutoff. We also assessed Cohen's *Kappa* statistic to determine the agreement between the standard BMI cutoffs and MUAC cutoffs estimated by the aforementioned process. SAS version 9.4 was used to conduct all statistical analyses. The confidence interval was set at 95%, and the significance level, at 5%.

## Results

In total, we had complete anthropometric information on 618 women; of which, 213 were adolescents, 212 were lactating mothers, and 193 belonged to the WIPP group. The mean age of the adolescent participants was 16 years (standard deviation [SD], 1.2), whereas that of lactating mothers and WIPP was 24 (SD, 4.6) and 25 (SD, 4.7) years, respectively. In all study groups, Hindus were an overwhelming majority, whereas in terms of caste, women from ‘other backward castes’ comprised about two-thirds of the participants. Approximately, four of five women lived in a non-pucca (not entirely built of brick) house, and about one-fifth of them had access to a flush toilet. In terms of education, the adolescent women fared much better than the other two groups; 58% of them completed more than eight years of school education as against less than 30% in rest of the groups. The socio-economic and sociodemographic characteristics of the study participants are presented in Table 1.

**Table 1:** Sociodemographic and anthropometric characteristics of the study participants. Women's nutrition study, Bihar, 2016.

Characteristics	Adolescents (N = 213)	Lactating mothers (N = 212)	Women in the interpregnancy period (N = 193)
	Percentage^a^	Percentage^a^	Percentage^a^
Religion			
Hindu	84.5	85.4	87.6
Muslim	15.5	14.6	11.9
Others	0.0	0.0	0.5
Caste			
Scheduled caste	18.3	20.3	21.8
Scheduled tribe	2.4	1.9	2.6
Other backward castes	64.3	67.0	63.7
Others/general caste	15.0	10.9	11.9
Type of family			
Nuclear family	64.3	50.5	59.6
Joint family	35.2	48.6	40.4
Type of house^b^			
Kaccha	23.9	26.4	25.9
Semi-pucca	56.8	59.9	56.5
Pucca	19.3	13.7	17.6
Source of drinking water			
Piped water (own/community tap)	4.3	1.9	2.6
Hand pump (own/community)	94.3	95.3	94.3
Others (dug well, pond, river, and so on)	1.4	2.8	3.1
Type of toilet			
Own flush toilet	21.1	17.9	17.1
Own pit toilet	8.5	6.1	7.8
Community/public toilet	0.5	0.5	0.0
No facility/open defecation	70.0	75.5	75.1
Education			
No formal education	0.0	1.82	2.0
Studied up to 8th standard	34.04	48.18	41.0
Studied higher than 8th standard	65.96	50.0	57.0
Husband's education^c^			
No formal education	0.0	0.7	0.0
Studied up to 8th standard	29.41	33.57	40.6
Studied higher than 8th standard	70.59	65.73	59.4
Family's asset index^d^			
1st tertile (low wealth)	23.47	33.49	32.12
2nd tertile (middle wealth)	36.62	35.85	35.75
3rd tertile (high wealth)	39.91	30.66	32.12
	Mean (±SD)	Mean (±SD)	Mean (±SD)
Age (years)	16 (1.2)	24 (4.6)	25 (4.7)
Weight (kg)	42 (5.6)	45 (7.5)	45 (7.9)
Height (cm)	150 (8.8)	149 (5.6)	150 (5.8)
Body mass index (kg/m^2^)	19 (8.7)	20 (2.9)	20 (3.2)
Mid-upper arm circumference (cm)	23 (2.2)	24 (3.0)	23 (3.9)

SD, standard deviation.

a Observations with missing values excluded as applicable.

b Type of house: ‘kaccha’—house made of mud, grass, bamboo, thatch, and other low-quality materials; ‘pucca’—structure made of brick; ‘semi-pucca’—any combination of the components of ‘kaccha’ and ‘pucca’ houses.

c Only for married adolescents (N = 27).

d Based on possession of 25 different household items.

**Fig. 1:**
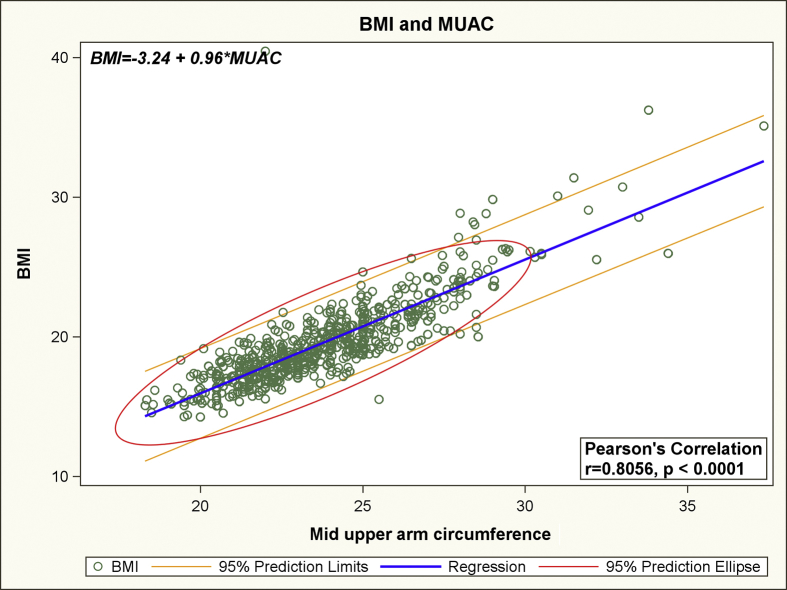
The linear relationship between Body Mass Index and Mid Upper Arm Circumference with 95% prediction limits. BMI, body mass index; MUAC, mid upper arm circumference.

**Fig. 2:**
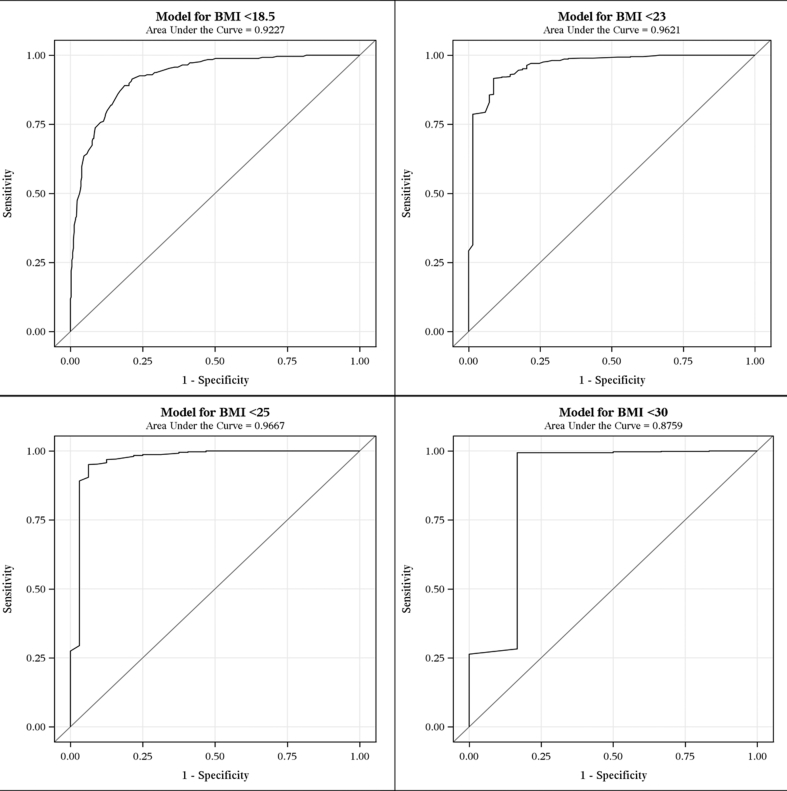
The area under the curve for the four different cutoffs for Body Mass Index. BMI, body mass index; MUAC, mid upper arm circumference.

Using Box–Cox power transformation for the simple linear regression model with BMI as the dependent variable and MUAC as the sole independent variable, we obtained a lambda value of 1.1 and an adjusted R-squared value of 0.74. Based on the lambda value of close to 1, it was determined that a linear relationship existed between BMI and MUAC.^32^ From the results of linear regression analysis, it was determined that the linear relationship between BMI and MUAC could be expressed by the following equation: *BMI* = -3.24 + 0.96*MUAC + ε (*P*-value for slope < 0.0001). Furthermore, a statistically significant positive relationship between BMI and MUAC was also established by a high Pearson's correlation coefficient (r = 0.81, *P* < 0.0001). The linear relationship between BMI and MUAC with 95% prediction limits is depicted in Fig. 1. In the ROC curve analysis, the area under the curve for the four different cutoffs for BMI ranged between 88% and 97% (Fig. 2). Based on the findings of ROC curve analysis, the MUAC cutoffs corresponding to the BMI cutoffs of 18.5, 23, 25, and 30 kg/m^2^ were estimated to be 23.2, 26.0, 27.3, and 30.5 kg/m^2^, respectively (Table 2). The extent of misclassification of the nutritional status of the study participants by using MUAC cutoffs instead of standard BMI cutoffs is presented in Table 3. Table 3 also depicts the sensitivity and specificity of MUAC cutoffs (considering BMI cutoffs as the gold standard) and the extent of agreement between different MUAC and BMI cutoffs. Other than the cutoff for ‘obese’ (BMI, 30 kg/m^2^), the *Kappa* coefficients for the rest of the MUAC cutoffs showed ‘substantial’ agreement (>0.6) with their BMI equivalents.^33^ The cutoff for ‘obese’ (*k* = 0.58) showed ‘moderate’ agreement with the corresponding BMI cutoff.

**Table 2:** MUAC cutoffs corresponding to BMI cutoffs, determined by different methods of ROC curve analysis. Women's nutrition study, Bihar, 2016.

Body mass index (kg/m^2^) cutoff	Mid-upper arm circumference (cm) cutoff (from different methods)		
	Youden's *J* statistic	Minimized distance to (0,1) point in the ROC curve	Sensitivity-specificity equality
18.5	23.2	23.2	23.0
23	26.0	26.0	26.0
25	27.3	27.3	27.0
30	30.5	30.5	26.0

ROC, receiver operating characteristic; MUAC, mid-upper arm circumference.

**Table 3:** Extent of misclassification on using different MUAC cutoffs as against the corresponding BMI cutoffs. Women's nutrition study, Bihar, 2016.

Body mass index (kg/m^2^)	Mid-upper arm circumference (cm)		Sensitivity and specificity of the chosen cutoff value	Total misclassification (%)	Kappa coefficient (95% CI)
	**<23.2**	**≥23.2**			
<18.5	193 (31.23)	53 (8.58)	0.89, 0.82	18.94	0.68 (0.62–0.73)
≥18.5	64 (10.36)	308 (49.84)			
	**<26**	**≥26**			
<23	494 (79.94)	59 (9.55)	0.91, 0.91	11.33	0.61 (0.53–0.69)
≥23	11 (1.78)	54 (8.74)			
	**<27.3**	**≥27.3**			
<25	565 (91.42)	24 (3.88)	0.95, 0.94	4.85	0.60 (0.48–0.72)
≥25	6 (0.97)	23 (3.72)			
	**<30.5**	**≥30.5**			
<30	608 (98.38)	4 (0.65)	0.99, 0.83	0.97	0.58 (0.30–0.86)
≥30	2 (0.32)	4 (0.65)			

MUAC, mid-upper arm circumference; BMI, body mass index; CI, confidence interval.

## Discussion

As per the fourth iteration of the National Family Health Survey (NFHS-4), about 32% of the 15- to 49-year-old women in rural Bihar were underweight.^34^ Therefore, addressing the issue of undernutrition among women in this state is a public health priority. Furthermore, obesity is slowly becoming a public health problem in India, though it is yet to reach the magnitude of developed world.^35^ Prior studies reported that obesity is not only more prevalent among women of reproductive age, compared to males, but also increasing at a faster rate among women.^36^ Therefore, the nutritional problems among Indian women are bifold.^37^ Assessment of nutritional status of adolescent and adult women is thus essential to determine the extent of this problem and to track the effectiveness of different measures to address malnutrition in this demographic stratum.

The present study collected anthropometric data on adolescent and adult women of rural Bihar, a socially and economically less developed state in India, and found that not only BMI and MUAC are strongly correlated but also a linear relationship exists between the anthropometric parameters. This corroborates the findings from earlier studies conducted in neighboring states of West Bengal and Jharkhand and other South-East Asian countries.^11^^,^^16^^,^^20^^,^^38^ Rather than the actual BMI value, public health programs tend to rely more on different BMI cutoffs as they allow for easier decision-making regarding intervention. Therefore, current analysis also focused on determining the MUAC cutoffs that corresponded to the standard BMI limits. The MUAC cutoffs determined using ROC curve analysis were found to have good agreement with BMI and showed excellent classification properties.

Our results therefore suggest that the MUAC cutoffs can serve as an effective screening tool for detection of malnutrition—both undernutrition and obesity—among women of Bihar and possibly other parts of India and neighboring countries with demographic characteristics similar to those of this impoverished Indian state. This constitutes an important finding from the public health nutrition perspective as MUAC is a far simpler measure, requiring less logistical (can be measured using inexpensive color-coded tapes) and intellectual (technical training) resources than BMI assessment (comparatively more sophisticated and difficult-to-carry instruments for weight and height measurement). A screening mechanism based on MUAC could be essential for early detection of malnutrition among women, who usually have poorer access to health care than men, and to bring them under care. Being a simpler measure, MUAC assessments can be implemented by frontline health workers such as AWWs and accredited social health activists (ASHAs) for community-level detection of malnutrition among women, especially in rural areas. The malnutrition cases detected this way can then be managed using simple community-based intervention under public health nutrition programs such as Integrated Child Development Services or, if found severe, by referral to health facilities. The present study also demonstrates the feasibility of measuring MUAC in study setting. To ensure compliance from rural women regarding anthropometric measurements, during enumerators' training, special emphasis was given on proper consent taking and explanation of the procedure to the study participants. The fact that there was no refusal toward measurement of MUAC by the study participants who provided consent for interview and anthropometric assessment, i.e., there was no refusal for MUAC measurement among the women who agreed to be interviewed, provides evidence that MUAC of rural women can be successfully measured by properly trained enumerators without much difficulty.

The current assessment suffered from a few limitations. Although the study had representation of women from across Bihar, a few important categories of women might have been left out. As the survey participants had to respond to a detailed interview and undergo anthropometric measurements, the probability of participation of sick women (who were also more likely to be malnourished) was low. Thus, the cutoff determined in the present study might have been based on the measurements taken from relatively healthier women, which might not comprehensively represent the scenario in the community. Exclusion of malnourished women would mean that at the population level, the sensitivity for screening of overweight and obese women might be lower, whereas that for underweight women would be higher than that obtained in the current analysis. As the burden of underweight among women of rural Bihar is much higher than that of overweight, from the perspective of public health nutrition programs, the measurement error might not be of much significance. Another obvious limitation of the present study is that because of its cross-sectional design, the health outcomes among women belonging to various anthropometric categories could not be assessed. Thus, we may not comment on whether the MUAC cutoffs can successfully identify women who are at higher risk of negative health outcomes. Furthermore, the extent of misclassification is the highest for the underweight cutoff (18.5 kg/m^2^). However, even for this cutoff, sensitivity of the corresponding MUAC cutoff for diagnosis of underweight is much higher than specificity. Therefore, despite some misclassification, being an easier measure and given the emphasis of the current nutrition program on identifying the underweight (even at the cost of some overdiagnosis), it may still be beneficial to the program. Finally, as the present study recruited women from rural Bihar, replicability of the cutoffs in urban settings and in other Indian states with varying sociodemographic characteristics remains to be evaluated.

The limitations notwithstanding, the present study is the first in India to assess the MUAC cutoffs corresponding to the standard BMI limits from a large and representative sample of women. The results suggest that it is possible to conduct community-level screening of malnourishment among adult/adolescent women using less resource-intensive techniques such as MUAC. Nevertheless, further work would be essential to estimate the MUAC standards for more granular age and physiological categories among women. In addition, longitudinal studies to understand the causal association between different MUAC levels and health outcomes would be immensely beneficial for nutrition programs.

## Author statements

### Acknowledgments

The authors are grateful to the study participants for their time in engaging with this study. They would also like to acknowledge the administrative support provided by State Health Society, Government of Bihar, India.

### Ethical approval

The present study was approved by the Ashirwad Ethics Committee, Ashirwad Hospital and Research Center, Ulhasnagar, India (ashirwadethicscommittee@gmail.com). Informed consent was collected from each agreeing participant before the interview and measurements, after explaining the details of the study in a language that the participant could understand.

### Funding

The study was funded by Bill and Melinda Gates Foundation (grant ID# OPP1084426). The funders had no role in study design, data collection and analysis, decision to publish, or preparation of the manuscript.

### Competing interests

The authors declare that they have no competing interests to disclose.

## References

[1] Kennedy E.T. (2006). Evidence for nutritional benefits in prolonging wellness. Am J Clin Nutr.

[2] Leandro-Merhi V.A., de Aquino J.L. (2014). Determinants of malnutrition and post-operative complications in hospitalized surgical patients. J Health Popul Nutr.

[3] Green C. (1999). Existence, causes and consequences of disease-related malnutrition in the hospital and the community, and clinical and financial benefits of nutritional intervention. Clin Nutr.

[4] Luma H.N., Eloumou S., Mboligong F.N., Temfack E., Donfack O.T., Doualla M.S. (2017). Malnutrition in patients admitted to the medical wards of the Douala General Hospital: a cross-sectional study. BMC Res Notes.

[5] Islam M.Z., Akhtaruzzaman M., Lamberg-Allardt C. (2004). Nutritional status of women in Bangladesh: comparison of energy intake and nutritional status of a low income rural group with a high income urban group. Asia Pac J Clin Nutr.

[6] Bhan N., Rao K.D., Kachwaha S. (2016). Health inequalities research in India: a review of trends and themes in the literature since the 1990s. Int J Equity Health.

[7] Woodruff B.A., Duffield A. (2002). Anthropometric assessment of nutritional status in adolescent populations in humanitarian emergencies. Eur J Clin Nutr.

[8] Laskey M.A. (1996). Dual-energy X-ray absorptiometry and body composition. Nutrition.

[9] Kyle U.G., Genton L., Pichard C. (2002). Body composition: what's new?. Curr Opin Clin Nutr Metab Care.

[10] Sánchez-García S., García-Peña C., Duque-López M.X., Juárez-Cedillo T., Cortés-Núñez A.R., Reyes-Beaman S. (2007). Anthropometric measures and nutritional status in a healthy elderly population. BMC Public Health.

[11] Nguyen P., Ramakrishnan U., Katz B., Gonzalez-Casanova I., Lowe A.E., Nguyen H. (2014). Mid-upper-arm and calf circumferences are useful predictors of underweight in women of reproductive age in northern Vietnam. Food Nutr Bull.

[12] Lee R., Nieman D. (2003). Nutritional assessment.

[13] Nube M., Asenso-Okyere W.K., van den Boom G.J. (1998). Body mass index as indicator of standard of living in developing countries. Eur J Clin Nutr.

[14] Shetty P.S., James W.P. (1994). Body mass index. A measure of chronic energy deficiency in adults. FAO Food Nutr Pap.

[15] FAO (July 29, 2017). Defining chronic energy deficiency. http://www.fao.org/docrep/T1970E/t1970e02.htm#P67_15391.

[16] Sultana T., Karim M.N., Ahmed T., Hossain M.I. (2015). Assessment of under nutrition of Bangladeshi adults using anthropometry: can body mass index be replaced by mid-upper-arm-circumference?. PLoS One.

[17] Ferro-Luzzi A., Sette S., Franklin M., James W.P. (1992). A simplified approach of assessing adult chronic energy deficiency. Eur J Clin Nutr.

[18] James W.P., Mascie-Taylor G.C., Norgan N.G., Bistrian B.R., Shetty P.S., Ferro-Luzzi A. (1994). The value of arm circumference measurements in assessing chronic energy deficiency in Third World adults. Eur J Clin Nutr.

[19] Briend A., Garenne M., Maire B., Fontaine O., Dieng K. (1989). Nutritional status, age and survival: the muscle mass hypothesis. Eur J Clin Nutr.

[20] Chakraborty R., Bose K., Koziel S. (2011). Use of mid-upper arm circumference in determining undernutrition and illness in rural adult Oraon men of Gumla District, Jharkhand, India. Rural Remote Health.

[21] Akhter N., Sondhya F.Y. (2013). Nutritional status of adolescents in Bangladesh: comparison of severe thinness status of a low-income family's adolescents between urban and rural Bangladesh. J Educ Health Promot.

[22] Chakraborty R., Bose K., Bisai S. (2009). Mid-upper arm circumference as a measure of nutritional status among adult Bengalee male slum dwellers of Kolkata, India: relationship with self reported morbidity. Anthropol Anzeiger.

[23] Wijnhoven H.A., van Bokhorst-de van der Schueren M.A., Heymans M.W., de Vet H.C., Kruizenga H.M., Twisk J.W. (2010). Low mid-upper arm circumference, calf circumference, and body mass index and mortality in older persons. J Gerontol Ser A, Biol Sci Med Sci.

[24] National Family Health Survey (NFHS-4) (2015-16 2017).

[25] Das A., Mahapatra S., Sai Mala G., Chaudhuri I., Mahapatra T. (2016). Association of frontline worker-provided services with change in block-level complementary feeding indicators: an ecological analysis from Bihar, India. PLoS One.

[26] Das A., Chatterjee R., Karthick M., Mahapatra T., Chaudhuri I. (2016). The influence of seasonality and community-based health worker provided counselling on exclusive breastfeeding - findings from a cross-sectional survey in India. PLoS One.

[27] (2004). Appropriate body-mass index for Asian populations and its implications for policy and intervention strategies. Lancet.

[28] Youden W.J. (1950). Index for rating diagnostic tests. Cancer.

[29] Pandey M., Jain A. (2016). ROC Curve: making way for correct diagnosis2016. https://www.pharmasug.org/proceedings/2016/SP/PharmaSUG-2016-SP11.pdf.

[30] Reiser B. (2000). Measuring the effectiveness of diagnostic markers in the presence of measurement error through the use of ROC curves. Stat Med.

[31] Faraggi D. (2000). The effect of random measurement error on receiver operating characteristic (ROC) curves. Stat Med.

[32] Box G.E., Cox D.R. (1964). An analysis of transformations. J R Stat Soc Ser B.

[33] Landis J.R., Koch G.G. (1977). The measurement of observer agreement for categorical data. Biometrics.

[34] (2016). State fact sheet: Bihar; national family health survey 4.

[35] Arnold F, Parasuraman S, Arokiasamy P, Kothari M. Nutrition in India. National Family Health Survey (NFHS-3) India 2005-062009.,

[36] Gouda J., Prusty R.K. (2014). Overweight and obesity among women by economic stratum in urban India. J Health Popul Nutr.

[37] Kennedy G., Nantel G., Shetty P. (2006). Assessment of the double burden of malnutrition in six case study countries.

[38] Chakraborty R., Bose K., Bisai S. (2009). Mid-upper arm circumference as a measure of nutritional status among adult Bengalee male slum dwellers of Kolkata, India: relationship with self reported morbidity. Anthropol Anzeiger.

